# Efficacy and Safety of Tranexamic Acid in Control of Bleeding Following TKR: A Randomized Clinical Trial

**Published:** 2009-12

**Authors:** P N Kakar, Nishkarsh Gupta, Pradeep Govil, Vikram Shah

**Affiliations:** 1 Head of the Department of Anesthesia, Fortis Hospital, Shalimar Bagh, New Delhi; 2 Attending Consultant, Department of Anesthesiology and Pain Management, Max Super specialty Hospital, Saket, New Delhi. 110017; 3 Associate consultant, Department of Anesthesiology and Pain Management, Max Super specialty Hospital, Saket, New Delhi. 110017; 4 Ex Senior resident, Department of Anesthesiology and Pain Management, Max Super specialty Hospital, Saket, New Delhi. 110017

**Keywords:** Tranexamic acid, TKR, blood loss

## Abstract

**Summary:**

Total knee arthroplasty (TKA) is generally carried out using a tourniquet and blood loss occurring mainly post operatively is collected in drains. Tranexamic acid is an antifibrinolytic agent which decreases the total blood loss. Patients had unilateral / bilateral cemented TKA using combined spinal and epidural anaesthesia. In a double-blind fashion, they received either placebo (n=25) or tranexamic acid (n=25)10 mg.kg^−1^ i.v., just before tourniquet inflation, followed by 1 mg kg^−1^ h-1 i.v. till closure of the wound. The postoperative blood loss, transfusion requirement, cost effectiveness and complications were noted. The groups had similar characteristics. The mean volume of drainage fluid was 270 ml and 620 ml for unilateral(U/L) and bilateral(B/L) TKR patients in placebo group. Whereas it was 160ml and 286 ml respectively in unilateral(U/L) and bilateral(B/L) TKR patients who received tranexamic acid. This was considered statistically significant. Control group patients received 26 units of PRBC as compared to 4 units in tranexamic acid groups (*p*<0.001). This was again statistically significant. None of the patients in any of the groups developed deep vein thrombosis. Tranexamic acid decreased total blood loss by nearly 54% in B/L TKR and 40% in U/L TKR and drastically reduced (> 80%) blood transfusion.

## Introduction

Major orthopedic procedures including hip and knee replacement and spine surgery, are associated with severe bleeding because of extensive dissections through bony and fibrotic tissue, increased fibrinolysis due to tourniquet application and surgery and inability to cauterize bleeding bony surfaces.^1^,^2^

Tissue and vascular damage during surgery or trauma, stimulates cascade of coagulation leading to clot formation to prevent blood loss. However during surgery and trauma the fibrinolytic system is also activated which leads to premature breakdown of the clot and excessive blood loss. 3,4

The activation of plasminogen, the plasma precursor of the proteolytic enzyme plasmin mediates fibrinolysis. Plasminogen binds to lysine residues on the surface of fibrin and is converted to plasmin by an activator released from endothelial cells [tissue plasminogen activator (t-PA)] that simultaneously binds to fibrin. Plasmin then degrades fibrin into soluble fibrin degradation products. 3 Tranexamic acid is a synthetic derivative of the amino acidlysine, which exerts antifibrinolytic effect through reversible blockade of lysine binding sites on plasminogen molecules. By blocking lysinebinding sites on plasminogen molecules and thereby inhibiting the interaction of plasmin fibrin, it exerts its antifibrinolytic effect. 5,6

So, this study was designed to evaluate the efficacy of tranexamic acid (TAX) in reducing blood loss and postoperative blood transfusions following TKR.

## Methods

After Institutional, review board approval, a double blinded, prospective, randomized, placebo controlled study was performed in 50 patients undergoing primary cemented total knee arthoplasties (both unilateral(U/L) and bilateral(B/L).Written consent was obtained from all patients.

Patients were excluded if they had one of the following criteria: known or suspected allergy to medications used (TAX, local anaesthetics, midazolam, pethidine, propofol), inherited or acquired hemostatic diseases, abnormal coagulation screening tests (platelet count, prothrombin time, activated partial thromboplastin time), ingestion of aspirin or other nonsteroidal anti-inflammatory drugs within seven days of surgery, renal or hepatic insufficiency, pregnancy, history of deep venous thrombosis (DVT) or pulmonary embolism or history of ocular pathology or ophthalmological procedure other than corrective lenses.

Patients were randomly allocated, into four groups as follows:

Group TU (n = 12): U/L TKR patients received TAX

Group TB (n = 13): B/L TKR patients received TAX

Group CU (n = 12): U/L TKR patients received NS

Group CB (n = 13): B/L TKR patients received NS

All patients underwent a pre anaesthetic check up and were premedicated with oral ranitidine 150 mg, alprazolam 0.25 mg and metoclopramide 10 mg HS and in morning of surgery. All patients were monitored with five-lead electrocardiography (ECG), pulse oximetry; end tidal carbon dioxide, core temperature through nasal probe and non invasive blood pressure monitoring.

The anaesthetist, surgeon and the observer were blinded to the study drug. A person not further involved in the study prepared and started the test/placebo drug before tourniquet inflation. In Group TU and TB, Tranexamic acid was given immediately before inflation of the tourniquet. After a test dose of 1mL, patients received a dose of 10 mg.kg^–1^ IV followed by an infusion of 1 mg.kg^–1^1hr^–1^ until skin closure. Patients in Group CU and CB received an equivalent volume of physiologic saline. Pneumatic tourniquet around the upper thigh was inflated to a pressure of 250-300 mm Hg in all patients before incision and deflated at the end of surgery. Before the surgery, Hb transfusion trigger point was determined for each patient according to the following criteria: for patients over 60 yr and associated cardiopulmonary disease the transfusion trigger was 10 g dL^−1^ whereas for other patients, the transfusion trigger was 8 g dL^–1^.

Intraoperative blood losses were negligible because of tourniquet. Postoperative blood losses were assessed by measuring wound drainage until drains withdrawal (± 24 hr). During surgery and in postoperative period, measured blood losses were replaced with Ringer's lactate in a 3:1 ratio and/or with pentastarch 10% (maximum dose 1500 mL) in a 1:1 ratio until Hb concentration fell below the transfusion trigger point. Thereafter, patients received leuco depleted allogenic packed red blood cells. Factors known to influence intraoperative and postoperative blood losses were noted. These included tourniquet time and pressure, length of surgery, mean arterial blood pressure maintained during surgery and minimal core temperature achieved.

After surgery patients were shifted to post anaesthesia care unit for further management. Post operative pain was managed with epidural infusion of 0.125% bupivacaine with clonidine 1.5 mcg.ml @ 4-6 ml/hr. Hemoglobin concentration, platelet count; coagulation profile and renal function tests were measured in the immediate postoperative period, after 4 hours and on postoperative day one. Drains were removed after 24 hours in the postoperative period.

## Statistical analysis

For tests of differences between quantitative data, two-sided t-tests were used. In the text, data are presented as mean ± standard deviation (SD) and p values of <0.05 are considered significant.

## Results

There were no significant differences between the patients with respect to age, sex, weight, duration of surgery and fall in core temperature. (Table 1)(*p* > 0.05)

**Table 1:** Demographic profile

		Groups			
Variables	CB(13)	TB(13)	CU(12)	TU(12)
**Age(yrs)**	67.15 (6.9)	63.13 (16.8)	66.2(4.8)	62.4(9.4)
**Weight(kg)**	64.23 (9.7)	69.23 (11.2)	63.4 (7.2)	67.9 (10.8)
**Sex(M/F)**	3/10	4/9	4/8	3/9
**Duration of surgery(min)**	152 (17.3)	154 (11.5)	92.1 (10.8)	96.8 (17.7)
**Temp(°C)**
Initial	36.2(0.9)	34.7(0.9)	36.4(0.9)	34.8(1.2)
Final	36.3(0.8)	34.7(1.1)	36.6(1.3)	34.8(1.4)

Data Mean (standard deviation), #*p*< 0.05

**Fig 1:**
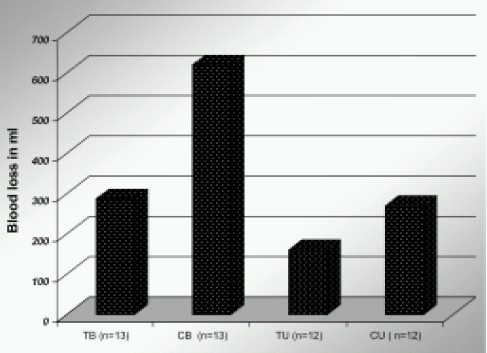
Effect of TAX on Postoperative Blood loss

The mean total blood loss was 270 ±88 ml and 620± 75 ml in U/L and B/L TKR patients in control groups. Whereas, in groups TU and TB (Those who received TAX), blood loss was 160 ± 87 ml and 286 ± 83 ml respectively. (P< 0.05) (Fig. 1) This was statistically significant.

**Fig 2:**
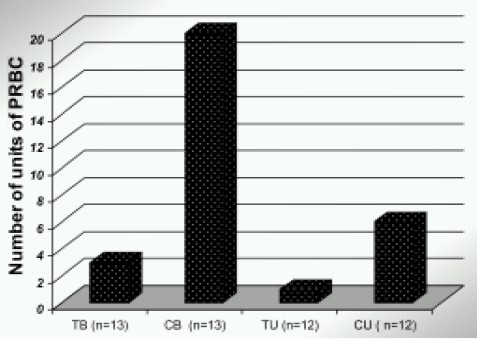
Effect of TAX on Postoperative blood transfusions

**Fig 3:**
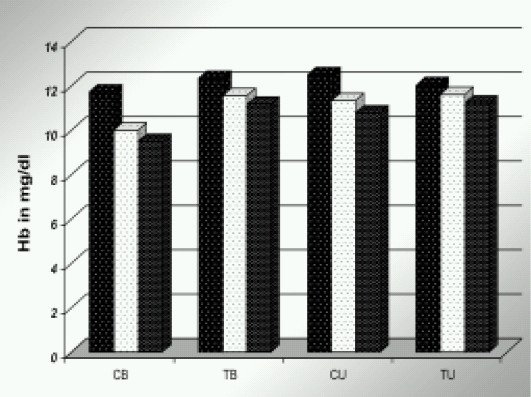
Effect of TAX on postoperative Hb concentration

Altogether, the control group was given 26 units, compared with 4 units in the tranexamic acid group (Fig.2.) Fall in hemoglobin concentration was considerably higher in the control group**.** (Fig 3) (*p* >0.05) This again was statistically significant.

None of the patients in any of the groups had clinical evidence of deep vein thrombosis observed daily for seven days or biochemical evidence of coagulation abnormality. Intraoperative hypotension requiring treat ment was comparable in the two groups.

## Discussion

We have investigated the effect of intraoperative tranexamic acid on blood loss and reduction of blood transfusion required after total knee arthoplasties.

Tranexamic acid (1, 4- amino- carboxylic acid) is an antifibrinolytic agent which is seven to 10 times as potent as epsilon aminocaproic acid (EACA). The volume of distribution is 1.0 litre kg^-1.^ and the plasma half-life approximately 80 min. The therapeutic concentration is 5-10 mg.lit^-1.^ The therapeutic effect of tranexamic acid is apparent when the haemostatic system has produced a fibrin clot which is prematurely dissolved by the proteolytic action of plasmin. 7

We had given tranexamic acid before inflation of tourniquet because fibrinolytic activation is a cascade process that is most easily inhibited in its earlier phases.

Moreover, previous research on tranexamic acid and thrombosis has failed to show any thrombogenic effect, even in patients who were treated for several days or even weeks.^4^,^8^ This may be due to the fact that fibrinolytic activity in vein walls is not affected by tranexamic acid. 4,8 We did not do routine screening for thrombosis in our study because all the patients received LMWH in the postoperative period.

As shown in numerous studies, the fibrinolytic response after trauma is biphasic with an increased activity during the first hours, followed by a shutdown that peaks at about 24 hours. 9 After knee arthroplasty the early post-traumatic fibrinolysis is further augmented by that induced by the tourniquet.^10^ The dosage regimen adopted by us seems to be an adequate compromise between fibrinolytic inhibition and the risk of inducing an augmented fibrinolytic response.

In our study there was reduction in blood loss by 40% in Group TU and 54% in Group TB (patients receiving tranexamic acid). (Fig 1) This reduction in blood loss was statistically significant. This also reduced the postoperative blood transfusions by > 80%.(Fig. 2) which again was statistically significant. The higher effectiveness in B/L TKR patients can be explained by the fact that in B/L TKR patients the bleeding is more as fibrinolytic system is already activated when second joint is operated.

The results are comparable to Cochrane review on “antifibrinolytic use for minimizing perioperative blood transfusion”. It included 21 trials of tranexamic acid vs. control (hip and knee replacement), and reviewed 993 patients in orthopedic surgery. It showed that tranexamic acid, significantly reduced allogenic blood transfusion (56% ) and total amount of blood lost during perioperative period (avg. 440 ml) in orthopedic surgery. 11

Beoni et al also showed a 48% reduction in blood loss and 70 % reduction in postoperative blood transfusion with the use of tranexamic acid. The total number of transfused units was 12 in the prophylactic group of 43 patients as against 40 in the placebo group of 43 (p = 0.002).^4^

Patients in the tranexamic acid group were given 4 units of blood in total, compared with 26 units in the control group. In our hospital the dose of tranexamic acid given would cost Rs. 166, compared with Rs. 6000 for a unit of leucodepleted banked blood. Thus, the immediate saving in the patients given tranexamic acid would have been about Rs. 5000. **(Cost calculation in appendix)**

**Appendix:** 

•Cost of 1 unit leuco depleted PRBC = Rs. 6000
•Total cost of blood in Control patients ( 26 units) = Rs 1,56,000
•Total cost of blood in TAX patients ( 4 units) = Rs 24, 000
•Cost of 1 ampoule of TXA = Rs 166
•Cost of TXA ( 25 patients) = Rs 8,300
•Cost of blood saved by giving TXA= Rs 1,23,700
•Cost saved per patient = Rs 4958
•Potential savings per year ( 500 patients) = Rs 25,00,000

To our knowledge, giving tranexamic acid is the only blood saving method that is cheaper, per saved unit, than banked blood in this type of surgery. This estimate does not include potential adverse effects from banked blood such as immediate transfusion reactions, transmission of infectious agents and disturbances of the immune system.

In conclusion, TXA reduces perioperative bleeding by almost a half in patients undergoing TKR, and reducing blood transfusion requirement in these patients by almost 80%.It is also tempting to determine if additional benefit could be achieved by repeating the Tranexamic acid in the post operative period.
